# Chitosan‐Coated Edaravone‐N‐Benzyl Pyridium Hybrid Polycaprolactone/ Poloxamer 188 Nanoparticles for Enhanced Alzheimer's Disease Drug Delivery

**DOI:** 10.1002/alz70859_106679

**Published:** 2025-12-26

**Authors:** Teboho E Tutubala, UWC Pharm Chem, Dubelab

**Affiliations:** ^1^ SAMRC, Cape Town, Western Cape South Africa

## Abstract

**Background:**

Alzheimer's disease (AD) presents a significant challenge due to limited treatment options that only provide symptomatic relief. A promising approach is the development of multifunctional agents capable of halting AD's degenerative processes. One such agent, the edaravone‐N‐benzyl pyridium hybrid (EBPD), designed by our group, has demonstrated neuroprotective properties. However, EBPD faces solubility, stability, and blood‐brain barrier (BBB) permeability issues. To address these challenges, this study aims to synthesize and characterize EBPD encapsulated in chitosan (CS)‐coated polycaprolactone (PCL) and Poloxamer 188 (P188) nanoparticles (NPs), aiming to enhance EBPD stability, solubility, and BBB permeability for AD treatment.

**Method:**

PCL‐EBPD and P188‐EBPD CS‐coated NPs were prepared using emulsion‐solvent evaporation and self‐assembly methods, respectively, and characterized for size, polydispersity index(PDI), and zeta potential using Dynamic Light Scattering (DLS) and Scanning Electron Microscopy (SEM), High‐performance liquid chromatography (HPLC) was used to determine drug concentration, drug load, and encapsulation efficiency. Further characterization, including chemical interactions, thermal stability, and crystallinity, was performed using Fourier Transform Infrared Spectroscopy (FTIR), Differential Scanning Calorimetry (DSC), and Powder X‐ray Diffraction (PXRD), respectively.

**Result:**

DLS analysis revealed PCL‐EBPD‐CS and P188‐EBPD‐CS NPs had sizes of 400‐600 nm, PDI values of 0.154‐0.3, and zeta potentials of +15 ‐ +17 mV, indicating uniform distribution, stability, and potential for enhanced BBB penetration. SEM analysis confirms smooth, uniform surfaces, suggesting reduced immune recognition and efficient encapsulation. HPLC analysis confirm EBPD 's tautomeric nature, while FTIR analysis showed successful encapsulation without chemical interactions. DSC analysis indicated thermal stability and PXRD analysis confirmed the amorphous nature of encapsulated EPD, with changes in the angle θ supporting disrupted crystallinity.

**Conclusion:**

In conclusion, NPs were successfully synthesized and characterized, demonstrating stability, efficient EBPD encapsulation, and potential for BBB permeation. Future studies will evaluate drug release, loading efficiency, cytotoxicity, and BBB permeability using bend5 cells to validate their therapeutic potential for AD treatment.

